# Case Report: Comprehensive management of bedside ligation of patent ductus arteriosus in an extremely low birth weight infant

**DOI:** 10.3389/fcvm.2026.1824677

**Published:** 2026-05-19

**Authors:** Juan Chen, Yu Luo, Maojun Li, Qian Yang, Rong Huang, Binzhi Tang

**Affiliations:** 1 Department of Pediatrics, School of Medicine and Life Science of Chengdu University of Traditional Chinese Medicine, Chengdu, China; 2 Department of Pediatrics, Sichuan Provincial People’s Hospital, School of Medicine, University of Electronic Science and Technology of China, Chengdu, China

**Keywords:** bedside ligation, comprehensive management, extremely low birth weight infants, literature review, patent ductus arteriosus

## Abstract

Patent ductus arteriosus (PDA) that fails to close after birth can lead to a hemodynamically significant left to right shunt, resulting in pulmonary overcirculation and systemic hypoperfusion. Currently, ligation performed in the operating room or at the bedside in the neonatal intensive care unit (NICU) are options for surgical treatment of hsPDA that has failed to close following pharmacological intervention, and bedside ligation is considered an optimized approach for preterm and extremely low birth weight (ELBW) infants. However, numerous challenges regarding the treatment of neonatal hsPDA via bedside ligation remain unsolved. Recently, a successful bedside PDA ligation has been performed in the NICU of Sichuan Provincial People's Hospital to treat an ELBW neonate with a gestational age of 27 + 1 weeks and a birth weight (BW) of 740 g. Echocardiography conducted 2 h after ligation revealed no distinct residual shunt at the great artery level. The infant's overall condition was favorable, and was successfully weaned from invasive mechanical ventilation on postoperative day 4. This article focuses on the diagnosis and comprehensive management of hsPDA in the presented case, synthesizing the findings with an extensive literature review to serve as a reference for managing such newborns with hsPDA.

## 1 Introduction

Following birth, with the ligation of the umbilical cord and the establishment of spontaneous breathing, systemic vascular resistance increases while pulmonary vascular resistance decreases, and functional closure of the patent ductus arteriosus (PDA) typically occurs within approximately 10 to 15 h postpartum. However, in more than 50% of premature infants with a gestational age (GA) ≤ 28 weeks, the PDA remains patent (1). Among these infants, only 48% can close naturally, and the majority will develop hemodynamically significant patent ductus arteriosus (hsPDA), which is characterized by a large left-to-right shunt from the aorta to the pulmonary artery (2). This results in pulmonary overcirculation and systemic hypoperfusion, which may precipitate multiple complications and adversely affect clinical outcomes in neonates (3–5). For patients with hsPDA who have clear indications for closure, pharmacological therapy is considered the first-line treatment in clinical practice, and ibuprofen, acetaminophen, and indomethacin are most commonly prescribed (6). If pharmacological intervention proves ineffective, surgical ligation or transcatheter closure for hsPDA will be considered and bedside ligation was generally regarded more favorable for preterm and extremely low birth weight (ELBW) infants. Transcatheter closure is an alternative to surgical ligation for PDA in extremely low birth weight (ELBW) infants. Though use has increased recently—driven by advances in low-profile delivery systems and softer, miniaturized occluders, it remains controversial for ELBW infants (7),. The 2025 SCAI Position Statement notes high procedural success but insufficient long-term outcome data, plus risks in tricuspid valve injury, cardiac perforation, and late-onset vessel obstruction (7). A 2025 expert consensus similarly highlights its promise for severely ill, extremely premature infants with PDA, yet stresses that high-quality evidence on efficacy, safety and feasibility in this population remains lacking, contributing to considerable clinical uncertainty (1). Therefore, bedside PDA ligation still accounts for a relatively large proportion in clinical applications. However, few cases of bedside operation have been reported in developing regions such as southwest China despite continuous improvements in surgical techniques and nosocomial infection control. In this paper, we summarize the experience of managing ELBW neonate who underwent bedside PDA ligation. Additionally, we review the relevant literature to provide a comprehensive relevant reference for the perioperative management of neonatal bedside PDA ligation.

## 2 Case report

A preterm male with GA 271/7 week and birth weight (BW) 740 g, was delivered via cesarean section due to maternal severe preeclampsia. He appeared cyanotic and hypotonic, no crying, and a heart rate (HR) of 40 beats/min at birth. Following guideline (8), he was quickly moved to a preheating radiant warmer at 35℃, immediately intubated and connected to a T-piece resuscitator for positive-pressure ventilation. Subsequently, 200 mg/kg of pulmonary surfactant (PS) was intratracheally given and HR elevated to 130 beats/min and saturation of pulse oxygen (SpO2) to 94% at 10 min of age. The Apgar scores were 1 at 1 min, 7 at 5 min, and 8 at 10 min after birth. Following postnatal resuscitation, he was quickly transferred to the neonatal intensive care unit (NICU) for further treatment. Physical examination at NICU admission was: lethargy, poor response, weak spontaneous breathing, warm extremities with capillary refill time (CRT) 2 s, body temperature 36.6 °C, HR 134 beats/min, and artificial breath 60 times/min.

Circulatory support: Fluid management was optimized based on postnatal day (PND), fluid balance, and body weight. Upon admission, Laboratory results revealed elevated myocardial marker: high-sensitivity troponin T at 333.00 ng/L, Brain natriuretic peptide (BNP) 3,900.7 pg/mL. Consequently, an infusion of creatine phosphate infusion was administered to provide cardioprotection. At PND 3, the infant underwent an echocardiogram that revealed a bidirectional shunt, predominantly left-to-right, with a width of 3.1 mm at the level of the great arteries. Considering the significant PDA and clinical manifestations including oliguria, metabolic acidosis increased pulse pressure, and feeding intolerance, a diagnosis of hsPDA was established, necessitating closure. Oral ibuprofen has been regarded as the first-line drug for PDA closure owing to its reportedly lower risk of adverse reaction (9, 10). However, given this abnormal coagulation function and pulmonary hemorrhage, which contraindicate the use of ibuprofen, acetaminophen (15 mg/kg per dose, administered orally every 6 h) was initially employed to close the PDA for a duration of 7 days starting from PND 9. The first course of drug treatment failed to close the PDA. After the improvement of coagulation function, a second course of drug intervention was initiated on the 16th day after birth. The treatment plan was adjusted to oral ibuprofen, with an initial dose of 10 mg/kg, followed by a maintenance dose of 5 mg/kg, once every 24 h. Two attempts to close the patent ductus arteriosus (PDA) with medication failed. The hsPDA has a significant adverse impact on both the pulmonary and systemic circulation, necessitating urgent surgical ligation of the PDA. To this end, a multiple multidisciplinary team (MDT) has been convened to conduct comprehensive evaluations, and the perioperative management has been thoroughly discussed. After carefully considering the opinions of MDTs and thoroughly evaluating the advantages and disadvantages, neonatologists conclude that following two unsuccessful courses of standard drug treatment, the hsPDA still existed. This condition met the criteria for surgical ligation as indicated in reference (11) and there were no absolute contraindications for surgery. Given the significant major risks associated with displacement of catheters or endotracheal tube, the difficulty in maintaining warmth and hydration, fluctuations in HR, blood pressure, and oxygenation due to movement stimulation, and the potential for intracranial hemorrhage during the transfer from the NICU to the operating room, these conditions are particularly detrimental to ELBW infants. The parents were comprehensively informed of the infant's critical condition, the failure of non—surgical interventions, and the potential risks and benefits of bedside PDA ligation. After fully comprehending the situation, they adhered to the recommendation of multidisciplinary team and provided “written informed consent for the procedure”. It was decided to perform PDA ligation at the bedside, with strict adherence to protocols for cleaning, disinfection, and infection control, and a medical team comprising core staff from the NICU and surgical ICU assumed responsibility for postoperative management.

**Figure 1:**
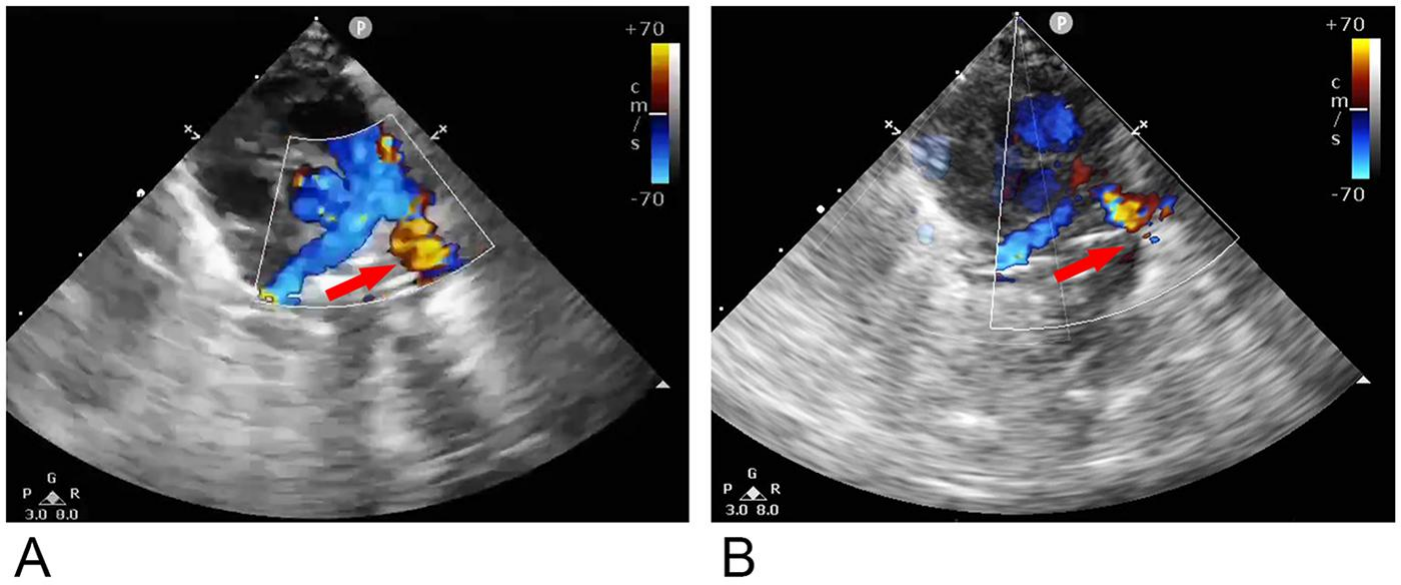
Echocardiography before and after PDA ligation. **(A)**: Echocardiography performed 1 day prior to surgery revealed color Doppler evidence of multicolored blood flow within the pulmonary artery deriving from the descending aorta (red arrow); **(B)**: Echocardiography conducted 2 h postoperatively demonstrated no residual shunt at the level of great artery (indicated by the red arrow).

Prior to the operation, the fluid intake was restricted to 85 mL/kg·d, the morning fasting serum cortisol level was measured at 7.43ug/dL, indicating normal adrenal function. Following comprehensive preoperative preparation, bedside PDA ligation was successfully performed at PND 26 (Table 1). During the surgical procedure, no significant adhesions were observed within the thoracic cavity. However, pulmonary congestion was seen in the left lung, and a tubular structure measuring approximately 5 mm in diameter and 4 mm in length was identified between the descending portion of the aortic arch and the pulmonary artery. A palpable tremor was noted at the pulmonary artery end, and the diameter of the aortic arch was approximately 3 mm. After ligation, the tremor at the pulmonary artery end disappeared, and pulmonary congestion markedly improved. As shown in Figure 1, echocardiography performed 2 h postoperatively revealed no significant residual shunt at the level of the great artery compared to preoperative findings, with an LVEF of 0.53. The specific postoperative assessment is shown in Table 1. At PND 30, he was successfully weaned from invasive ventilation and transitioned to non-invasive NIPPV mode.

**Table 1:** Timeline of clinical course and management.

PND	Key clinical events	Clinical, Laboratory, and Imaging Characteristics of the Patient	Therapeutic intervention
1	-	hs-cTnT 333.00 ng/L, BNP: 3,900.7 pg/mL. Blood gas analysis: pH 7.05, BE −12.5 mmol/L, Lac 4.3 mmol/L	Intravenous administration of creatine phosphate
1–10	Improvement of coagulation function	Pulmonary hemorrhage and coagulation abnormalities	PND1–4: Intravenous administration of hemostatic drugs. PND1, 2, 5, 8, 10: Intravenous administration of FFP, fibrinogen, and vitamin K1
3	Diagnosis of hsPDA	PDA with a width of 3.1 mm, bidirectional shunt predominantly left-to-right, at the level of the great artery. LA/AO：1.8. Clinical manifestations: oliguria, metabolic acidosis, increased pulse pressure, feeding intolerance	Expectant treatment
7	Failure in weaning off the invasive ventilator	-	-
9	The first course of medication (PND 9–16)	PDA with a width of 3.1 mm, bidirectional shunt predominantly left-to-right, at the level of the great artery (PND8)	Oral acetaminophen (15 mg/kg per dose, administered orally every 6 h)
12	Day 3 of treatment	PDA with a width of 2.6 mm, continuous left-to-right shunt at the level of the great artery, the ratio of the PDA diameter^2^/BW: 9.14 mm^2^/kg; EF:0.65.	Continue the course of treatment.
15	Failure in weaning off the invasive ventilator	-	-
16	The second course of medication (PND 16–19)	PDA with a width of 2.8 mm, continuous left-to-right shunt at the level of the great artery. Transductal peak systolic velocity:1.5 m/s; EF:0.67	Oral ibuprofen (10 mg/kg loading dose, followed by 5 mg/kg daily)
19	Day 3 of treatment	PDA with a width of 3.0 mm, continuous left-to-right shunt at the level of the great artery. Transductal peak systolic velocity:2.9 m/s; the ratio of the PDA diameter^2^/BW: 9.78 mm^2^/kg; LA/AO: 2.3	-
26 days, and 1 h	Surgical intervention	-	-
26 days, and 3 h	Postoperative assessment	Postoperative assessment at 2 h：no residual shunt at the level of the great arteries. BNP: 63.0 pg/mL; Blood gas analysis: pH 7.41, BE 4.6 mmol/L, Lac 0.5 mmol/L. CXR and LUS: No pneumothorax, pleural effusion or other conditions.Respiratory rate, heart rate, blood pressure and urine output were all in good condition. BP:52/30 mmHg; SpO_2_: 92%	Prophylactic milrinone for the prevention of PLCS (loading dose: 0.5 μg/kg/min, changed to maintenance dose: 0.2 μg/kg/min after 3 h)
30	Successful weaning off invasive mechanical ventilation	-	-

PND, postnatal day; hsPDA, hemodynamically significant patent ductus arteriosus; hs-cTnT, high-sensitivity cardiac troponin T; BNP, B-type natriuretic peptide; LA/Ao, left atrial-to-aortic root ratio; EF, ejection fraction; BW, birth weight; FFP, fresh frozen plasma; PLCS, post-ligation cardiac syndrome; BP, blood pressure; SpO₂, peripheral oxygen saturation; CXR, chest x-ray; LUS, lung ultrasound. Nonoperative management of PDA in the present case. Following two unsuccessful attempts at pharmacological closure of PDA, bedside PDA ligation was initiated at PND 26. Events are listed in chronological order by postnatal day. Echocardiographic data are presented where available. The surgical intervention (bedside PDA ligation) is highlighted in bold.

Respiratory support: 200 mg/kg of PS was intratracheally given at 5 min, 30 h and 69 h, respectively, after birth. The baby received 54 days of MV in total, including invasive MV for 30 days, non-invasive MV for 24 days, and nasal oxygen inhalation for 15 days, and then completely independent of ventilator/oxygen at PND 69. A short course of low-dose dexamethasone a randomized trial (DART) regimen was adopted for BPD treatment.

Neurodevelopmental support: a head ultrasound revealed bilateral ventricular dilation. Subsequent brain function monitoring detected occasional sharp waves; however, no limb convulsions were observed. The baby was administered anticonvulsant treatment with phenobarbital and levetiracetam oral solution, neurotrophic therapy with vitamin B6.

Infection control: recurrent infections were observed despite separated in a single ward and strict aseptic procedures, which may be attributed to immunocompromise of the baby, and prolonged MV. Antibiotic therapy included ceftazidime, penicillin sodium, meropenem, vancomycin, linezolid, and amikacin for bacterial infections, while fluconazole was used for antifungal prophylaxis. The antibiotic regimen was adjusted according to his metabolic acidosis status and inflammatory indicators.

The baby was discharged home 81 days after operation at post menstrual age (PMA) 42^3/7^ weeks with body weight of 2.99 kg and without any requirement of respiratory support or oxygen supply, and a full oral feeds (FOF) of 53 mL milk was given every 3 h There were no signs of respiratory distress, cyanosis, fever, vomiting, milk choking, screaming, or convulsions, and no abdominal distension was observed. Circulatory indicators including HR, blood pressure, perfusion index, and CRT were within normal limits. Now he is 13-month-old, with body weight of 9,000 g and height of 85 cm, can walk and speak simple words, describing a well physical, motor, and intelligence development, and recent echocardiography showed no abnormities.

Written informed consent was obtained from the patient for publication of this case report and any accompanying images.

## 3 Discussion

### 3.1 Adverse effects of hsPDA

Premature ELBW infants with hsPDA exhibit a reduced likelihood of spontaneous ductal closure and are at an increased risk of developing severe adverse outcomes, including BPD, NEC, and intraventricular hemorrhage (IVH). The prolonged presence of hsPDA is associated with a higher incidence of related complications such as BPD and consequent mortality (12).

### 3.2 Treatment of PDA

#### 3.2.1 Expectant treatment

Expectant treatment is a conservative strategy that avoids direct closure of the PDA through pharmacological or surgical interventions. This strategy primarily encompasses moderate respiratory support, optimal ventilation and oxygenation, and restricted fluid intake. It has been evidenced that SpO2 < 92% is an independent risk factor for the failure of PDA closure in very preterm infants (13). Furthermore, neonates with BW < 1,500 g exhibit a significantly increased risk of hsPDA when SpO2 < 90% (14). Therefore, moderate respiratory support can effectively maintain adequate ventilation and oxygenation in neonates, promote the contraction of smooth muscle in the PDA, and facilitate both the anatomical and functional closure of PDA. Studies have shown that moderate-duration respiratory support (MV < 10 days) does not elevate the risk of BPD in infants with PDA (15). A national survey in Italy revealed that 80% of NICUs adopt a strategy of reducing fluid intake to decrease pulmonary blood flow, thereby promoting spontaneous closure of PDA in premature infants (16). Initial fluid intake is typically maintained at 60–80 mL/kg per day, which ensures the fulfillment of basic physiological requirements and maintains homeostasis in premature infants (16).At present, the efficacy and long-term prognosis of expectant treatment are still controversial (17). For preterm infants with a GA > 28 weeks, the spontaneous closure rate of PDA is approximately 70%, leading to expectant treatment often being regarded as the preferred initial treatment strategy (18).

The optimal management strategy for hsPDA in extremely preterm infants remains controversial and a subject under ongoing investigation (19). Historically, early aggressive closure—pharmacological or surgical—was favored to prevent complications like pulmonary hemorrhage, bronchopulmonary dysplasia (BPD), intraventricular hemorrhage (IVH), and necrotizing enterocolitis (NEC) (20). However, recent randomized controlled trials have challenged this, suggesting that a conservative, “watch-and-wait” approach may yield non-inferior outcomes while avoiding potential risks of cyclooxygenase inhibitors and surgery (21, 22). A 2025 meta-analysis of >2,000 premature infants confirmed lower mortality and less moderate-to-severe BPD with conservative management (23). Still, some infants with large, hemodynamically significant shunts fail to close spontaneously and risk progressive organ injury (24). Accordingly, the 2024 European Consensus Guidelines recommend pharmacologic or surgical closure for persistent hsPDA in ventilator-dependent infants beyond day 7—especially with pulmonary hemorrhage or systemic hypoperfusion (25).

#### 3.2.2 Pharmacological intervention

For children with hsPDA, early pharmacological intervention is recommended to facilitate PDA closure. Commonly used drugs include cyclooxygenase inhibitors such as indomethacin, ibuprofen, and acetaminophen. The primary pharmacological mechanism involves inhibiting prostaglandin synthesis, which induces smooth muscle contraction in the PDA and promotes PDA closure (26). Several studies have demonstrated that the efficacy of indomethacin, ibuprofen, and acetaminophen is comparable; however, indomethacin may be associated with a higher incidence of adverse reaction (9, 10, 27). A multi-center cohort study conducted in the United States revealed significant variations in the treatment regimens for PDA among ELBW infants across different centers. These disparities may be attributed to differences in institutional rescue capabilities, regional economic conditions, and individual variations in drug tolerance (28). Consequently, for ELBW infants with hsPDA, it is imperative to consider not only the efficacy and safety of the drugs but also to comprehensively evaluate the individual characteristics of each infant.

Due to the abnormal coagulation function observed in this case, the use of indomethacin and ibuprofen is contraindicated. Therefore, we prioritized acetaminophen as the preferred pharmacological agent for PDA closure. However, the initial course of pharmacotherapy was unsuccessful.

Studies revealed that the failure rate of the initial drug-induced closure ranged from 25% to 40% (9, 10), which was associated with several factors including the absence of prenatal glucocorticoid administrations, early GA, and primary lung disease (29, 30). Following the improvement of coagulation function, ibuprofen was administered for the second course of treatment, unfortunately the PDA failed to close once again.

Studies have demonstrated that the closure rate of the second course of drug treatment for PDA ranges from 43% to 63% (31, 32).However, due to variations in administration routes, GA, and body weight of patients across different studies, direct comparisons of these data are unreasonable. Following two unsuccessful courses of standardized drug treatment, the baby continued to exhibit hsPDA, demanding surgical ligation. Consequently, it was decided to perform PDA ligation at the bedside in the NICU after a MDT discussion.

#### 3.2.3 Surgical ligation

In 1938, Boston Children ’s Hospital successfully performed the first PDA ligation on a 7-year-old children (33). Currently, PDA ligation is safe for very low birth weight infants. A study revealed that among 449 very low birth weight infants (BW < 1,500 g) who underwent PDA ligation, only 2 cases resulted in mortality, representing a rate of 0.4% (34). Several studies have demonstrated that surgical intervention within 3 weeks after birth significantly improves respiratory outcomes and nutritional status in very preterm infants, facilitating early tracheal extubation and FOF (35–38). In addition, early surgery can mitigate hsPDA-related complications such as severe BPD, NEC, IVH, and sepsis, and reduce the duration of hospitalization (39).

Concerning the location of surgery, the prevailing consensus and majority of studies advocate for performing PDA ligation at the bedside for neonates with a GA < 30 weeks and BW < 1,000 g (40–42). In the early 1980s, researchers initiated investigations into the feasibility and safety of performing bedside surgery for preterm infants (43). Prior to this, transporting very preterm infants to the operating room posed significant risks, including unstable vital signs during transport, intracranial hemorrhage due to blood pressure fluctuations caused by mechanical vibrations, tracheal intubation dislodgement, and challenges in maintaining appropriate temperature and humidity (44). The risk of acute hemodynamic deterioration is also heightened during the postoperative transport back to the NICU (44). A predictive scoring system for clinical complications associated with neonatal in-hospital transport revealed an incidence rate of 24.6% (45).Within this scoring system, GA ≤ 28 weeks and transfer destination to the operating room were assigned higher scores. This indicates that extremely premature infants or those being transported to the operating room have a higher likelihood of experiencing complications. In addition, during the transfer between the NICU and the operating room, the NICU team, anesthesiologists, operating room nurses, and surgeons must conduct continuous and meticulous handover procedures. Concurrently, a rigorous risk assessment is necessary. Additionally, it is essential to ensure that high-quality equipment and medications meeting the requirements for transfer are available, which undoubtedly constitutes a complex and resource-intensive process. In contrast, bedside surgery can effectively minimize potential omissions and associated risks in the handover process, reduce the consumption of medical resources, and ultimately alleviate the economic burden on pediatric patients.

**Figure 2:**
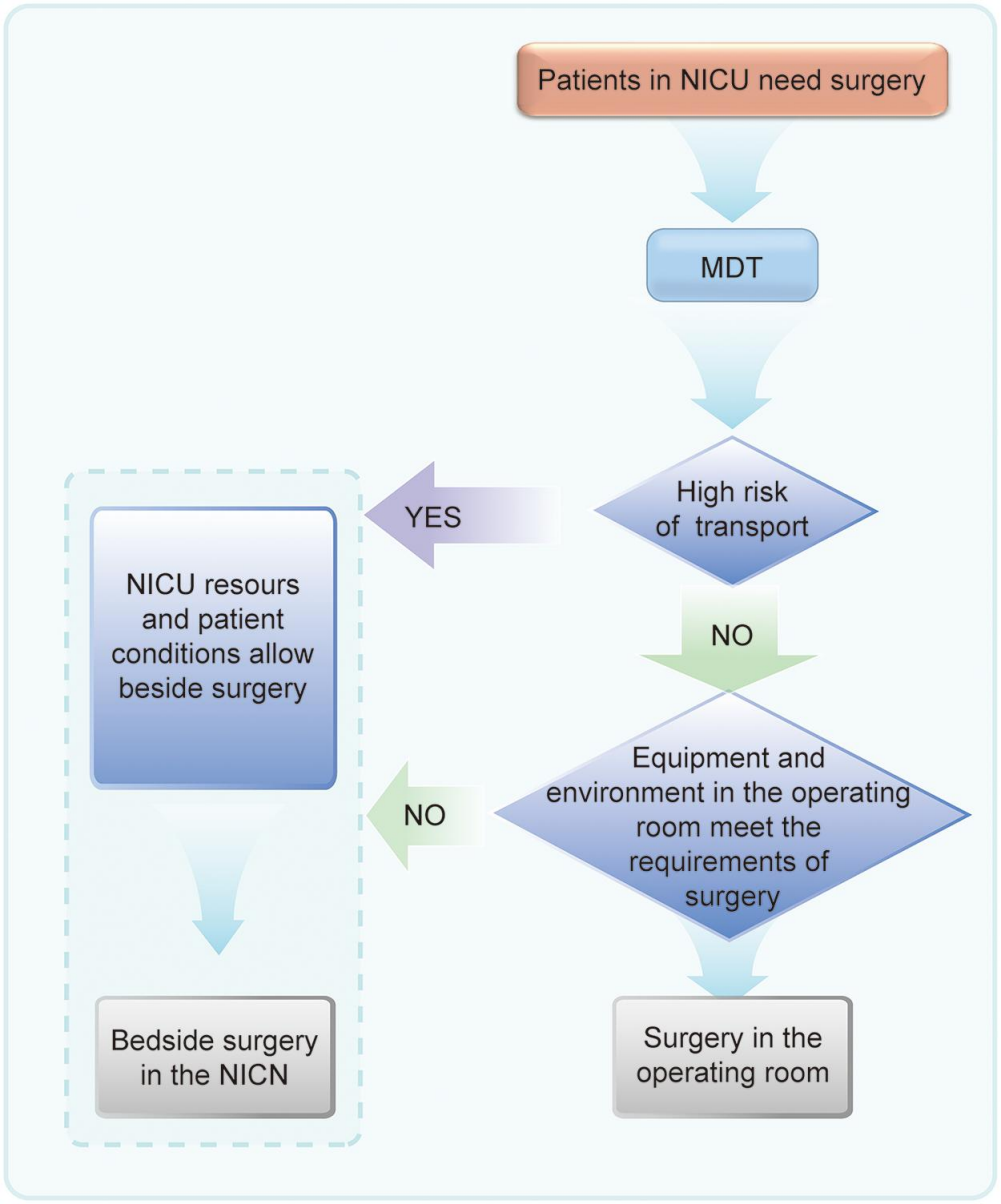
Place of surgery selection flow chart. This systematic approach offers a scientific basis for determining optimal place for neonatal surgery.

Of course, performing surgery at the bedside in the NICU is not without risks. The primary concern is that maintaining the aseptic conditions required for surgery can be challenging in the NICU environment, potentially increasing the risk of infection. However, advancements in ward disinfection and sterilization technologies have significantly enhanced the safety and feasibility of bedside surgery (46). In addition, the NICU exhibits certain limitations in comparison to the operating room with respect to space, equipment, and personnel. Specifically, the bed unit area in the NICU is more restricted, the lighting conditions do not comply with the specialized standards of the operating room, and most NICU nurses may not match the efficiency and accuracy of their counterparts in the operating room during surgery. Therefore, a decision flow chart, as illustrated in Figure 2, could be adopted for selecting place of operation for neonates in clinical practice, which facilitates a systematic evaluation of the advantages and disadvantages, thereby supporting evidence-based decision-making.

As early as 1986, Canada reported a successful case of bedside PDA ligation in the NICU (47). Since the beginning of the 21st century, the development of neonatal care disciplines in China has seen rapid advancement. Leading hospitals in more developed regions, including Peking Union Medical College Hospital, Shanghai Children's Hospital, and Children's Hospital Zhejiang University School of Medicine, have subsequently initiated NICU bedside surgery (48). Several tertiary hospitals in West China, such as the Children ’s Hospital of Chongqing Medical University, have successfully performed the first bedside PDA ligation in the past decade. However, due to various factors such as hospital reporting mechanisms and challenges in data collection and collation, it is difficult to obtain precise nationwide statistics on specific cases. Nonetheless, a review of relevant literature indicates that bedside PDA ligation surgery in China remains in its developmental stage, primarily conducted in top hospitals.

### 3.3 Perioperative management

#### 3.3.1 Management of post-ligation cardiac syndrome (PLCS)

Ligation surgery is an invasive procedure that carries the risk of adverse events, including but not limited to respiratory function deterioration, PLCS, and incision infections (49). Following PDA ligation, the left-to-right shunt suddenly ceased, leading to a reduction in the volume of blood returning to the left atrium. Meanwhile, the sudden increase in left ventricular afterload after ligation, coupled with the inability of the immature myocardium in premature infants to compensate, predisposes the patients to PLCS. The primary clinical manifestations include postoperative hypotension, inadequate tissue perfusion, and ventilation or oxygenation failure, which significantly increase the mortality among pediatric patients. A study conducted in France demonstrated that the incidence of PLCS in children weighing less than 2000g was approximately 15% (50). To prevent PLCS in the present case, the following comprehensive measures were implemented: a) Prior to the operation, the ventilation mode was switched from high-frequency oscillatory ventilation to constant frequency ventilation in order to stabilize the limb during operation. b) Preoperative evaluations, including a thorough assessment of adrenal function were actively performed, once he experience catecholamine-resistant hypotension postoperatively, early administration of low-dose hydrocortisone is adopted to prevent PLCS (51). c) During the surgical procedure, the child was positioned in a right lateral decubitus position to ensure stable respiratory function. A left axillary incision was used to ligate the PDA. The operation was meticulous and minimized stimulation to the heart and surrounding tissues. d) Postoperatively, left ventricular systolic function and relevant parameters were rigorously monitored and the immediate postoperative LVEF was measured at 53%. Consequently, milrinone was administered to enhance cardiac function and reduce afterload, with initial loading dose of 0.5 ug/kg·min, followed by a maintenance dose of 0.2ug/kg·min after 3 h. Milrinone is characterized by its vasodilatory properties and ability to augment myocardial contractility, and has been recognized as a biologically targeted drug for the prevention of PLCS (52). Owing to rigorous monitoring of postoperative cardiac function and the early proactive administration of milrinone, the baby's postoperative circulatory function remained relatively stable, and no significant PLCS was observed. The invasive ventilator was successfully weaned 4 days after the operation.

#### 3.3.2 Nosocomial infection control

Nosocomial infection is also a critical factor influencing postoperative survival rates in pediatric patients. Numerous studies have shown that factors such as GA < 30 weeks, BW < 1,500 g, the use of MV, central venous catheterization, and surgery significantly increase the risk of nosocomial infections (53–55). In addition, hsPDA itself was also considered to be associated with the occurrence of nosocomial infection (55, 56). To minimize the risk of infection during NICU surgery, the prevailing practice in China is to perform PDA ligation at the beside within the laminar flow clean ward of the NICU (46). Laminar flow clean ward has been implemented in the NICU of some major general hospitals and specialized children's hospitals in China, such as Eight-one Children's Hospital of Beijing Military General Hospital, Peking Union Medical College Hospital, Shanghai Children's Hospital, The Central Hospital of Wuhan and so on. However, due to constraints in funding, spatial limitations, medical requirements, and resource allocation, most NICUs, including ours, have not been equipped with laminar flow wards. To comply with the stringent requirements of a sterile environment for thoracotomy procedures, such as bedside PDA ligation, we implemented a variety of measures, including: a) All non-essential items within the surgical area should be removed before the operation. The surgical ward should undergo disinfection using an, air sterilizer combined with ultraviolet irradiation. Surface disinfection should be conducted with a 500 mg/L chlorine-containing solution, and environmental hygiene monitoring of the surgical room must be performed in accordance with the ‘Hospital Disinfection Hygiene Standard ‘ prior to the operation. b) Medical staffs underwent strict training in the prevention and control of nosocomial infection, ensuring stringent adherence to hand hygiene protocols and aseptic techniques. c) After the surgery, the children were placed in an isolation ward and assigned dedicated medical staffs to prevent cross infection and minimize the risk of contact transmission of pathogens. Postoperative infection indicators were monitored dynamically to facilitate early detection, prompt identification, and timely treatment of potential nosocomial infections.

#### 3.3.3 Take advantage of MDT

In response to the specific needs of the patient's condition, a MDT team was initially established, comprising specialists from the NICU, cardiac surgery, surgical ICU, anesthesiology department, nosocomial infection control office, and medical administration department the MDT team convened multiple meetings to deliberate on the necessity of surgery, the optimal timing, the location where the surgery carried out, and postoperative management plan at PND 22, PND 23, PND 24, PND 26, PND 27 and PND 28, respectively. Based on these discussions, a comprehensive treatment plan was formulated and subsequently implemented.

**Figure 3:**
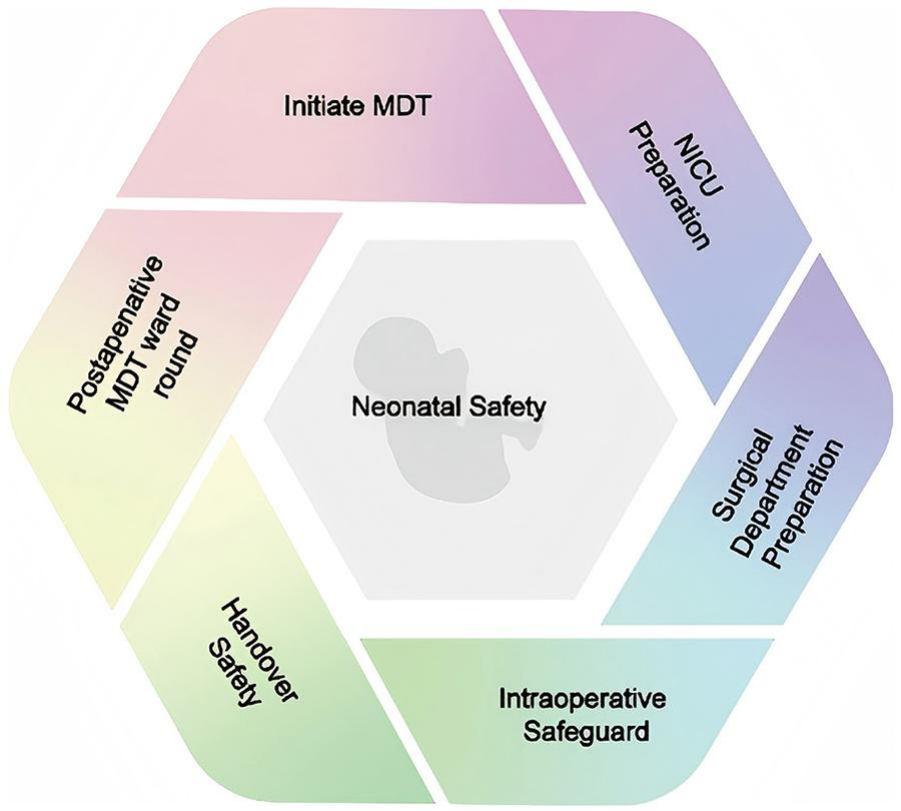
MDT collaboration process in perioperative management. Thisprocess has significantly enhanced the effectiveness of patient care and ensures optimal patient safety and therapeutic outcomes through a series of coordinated effort.

MDT refers to a multidisciplinary team comprising experts from more than two related medical disciplines. Through collaborative diagnosis and treatment discussions in a formal meeting format, the team formulates and implements the optimal treatment plan for patients (57). The Mayo Clinic in the United States first introduced and implemented the MDT model in the 1960s, initially focusing on its application in tumor diagnosis and treatment. By the 1990s, MDT was significantly refined and expanded across various medical fields through the efforts of medical centers such as Anderson (58). MDT started relatively late in China. The concept of the MDT model in general hospitals was first introduced in 2002 (59), and the first MDT team was established in Beijing Friendship Hospital. With the continuous integration with international standards, the utilization rate of MDT in major tertiary hospitals in China significantly improved. However, the overall coverage of MDT in China remains lower compared to global levels, predominantly limited to tertiary hospitals. According to data released by the National Health Commission of China as of 2018, 231 tertiary hospitals were implementing MDT, accounting for only 16% of all tertiary hospitals. This limited adoption may be attributed to the lack of standardized MDT guidelines and inadequate allocation of medical resources in certain tertiary hospitals. The implementation of MDT encompasses various facets of the healthcare system. To further improve the utilization rate of MDT in China, it is imperative to establish a comprehensive set of standardized national guidelines for MDT, encompassing aspects such as meeting frequency, participants, and discussion protocols. Additionally, government policy support and incentives are crucial, including financial subsidies and preferential policies for medical insurance reimbursement. Furthermore, there should be intensified efforts in promoting and educating about the MDT model to increase awareness and acceptance among healthcare providers, patients and the general public. Through the MDT, a comprehensive perioperative management plan for the baby and strategies to address various emergencies have been established, ensuring smooth surgical procedure and postoperative recovery. To facilitate understanding of the perioperative management and MDT collaboration process, this paper includes a relevant flow chart (Figure 3), which illustrates the critical stages of the perioperative period and highlights the pivotal role of MDT in surgical decision-making.

## 4 Conclusion

This paper reports the first case of successful bedside PDA ligation in the NICU at Sichuan Provincial People's Hospital, highlighting the institution's advantage of multidisciplinary collaboration as a general hospital. The patient was an ELBW infant with a GA of 271/7 weeks and a BW of 740 g. Following two unsuccessful attempts at pharmacological closure of PDA, a MDT concluded that bedside PDA ligation would be the most advantageous treatment option for the baby, provided stringent measures were in place to safeguard against nosocomial infections. Currently, there are few reports on bedside PDA ligation in China, and challenges such as nosocomial infections and postoperative complications remain significant. When determining the treatment strategy for infants with PDA, it is essential to comprehensively evaluate their clinical symptoms, signs, and echocardiographic results to select the optimal treatment, thereby improving patient's prognosis.

## Data Availability

The original contributions presented in the study are included in the article/supplementary material, further inquiries can be directed to the corresponding author.
